# Different Regimes of Opto-fluidics for Biological Manipulation

**DOI:** 10.3390/mi10120802

**Published:** 2019-11-21

**Authors:** John T. Winskas, Hao Wang, Arsenii Zhdanov, Surya Cheemalapati, Andrew Deonarine, Sandy Westerheide, Anna Pyayt

**Affiliations:** 1 Department of Chemical and Biomedical Engineering, University of South Florida, 4202 E. Fowler Ave. ENB118, Tampa, FL 33620, USA; John.Winsk@gmail.com (J.T.W.); wanghao@usf.edu (H.W.); arzhdanov@gmail.com (A.Z.); scheemalapati@usf.edu (S.C.); 2 Department of Cell Biology, Microbiology and Molecular Biology, University of South Florida, 4202 E. Fowler Ave. ENB118, Tampa, FL 33620, USA; deonarine@mail.usf.edu (A.D.); Westerheide@usf.edu (S.W.)

**Keywords:** opto-fluidics, micro-manipulation, cells, microparticles

## Abstract

Metallic structures can be used for the localized heating of fluid and the controlled generation of microfluidic currents. Carefully designed currents can move and trap small particles and cells. Here we demonstrate a new bi-metallic substrate that allows much more powerful micro-scale manipulation. We show that there are multiple regimes of opto-fluidic manipulation that can be controlled by an external laser power. While the lowest power does not affect even small objects, medium power can be used for efficiently capturing and trapping particles and cells. Finally, the high-power regime can be used for 3D levitation that, for the first time, has been demonstrated in this paper. Additionally, we demonstrate opto-fluidic manipulation for an extraordinarily dynamic range of masses extending eight orders of magnitude: from 80 fg nano-wires to 5.4 µg live worms.

## 1. Introduction

Until now, to the best of our knowledge, the most common and reproducible methods of microscale manipulation have included mechanical and magnetic micro-manipulators [1,2] and various types of optical tweezers [3] including: holographic [4], plasmonic [5], antenna-based [6], and photonic crystal-based tweezers [7]. While these methods work well on smaller particles and nano-scale objects [8,9], they are limited in their application to biological specimens. They might induce severe heating of biological material [10] and phototoxicity [7]. More recently, magnetic levitation of cells has allowed for 3D manipulation of biological specimen and sensing capabilities that were unachievable using traditional 2D manipulation of cells, however it required the use of special magnetic fluids that are not compatible with many biological applications [2]. 

An alternative approach to cell manipulation is to use optofluidic tweezers (OFT) in capillaries [6] or thermally-induced current generation by absorption in amorphous silicon [11] using a light source focused through a microscope objective. These approaches are fundamentally different from the traditional optical and optoelectronic tweezers, and magnetic levitation, as they can attain much stronger forces [12,13]. Most recently, a thermo-plasmonic approach to OFT has appeared as the next generation of micro-manipulation techniques. In plasmon-assisted microfluidics, plasmonic heating is used to generate convective flow patterns [14,15,16,17,18,19,20]. Initially, simulations included the modeling of convection currents induced by photo-heating a single gold disk [14], which predicted very low fluid velocities in nm/s range. Later, it was demonstrated that localized heating of plasmonic structures could produce theoretically predicted toroid-shaped convection patterns [14,15,16]. Consequently, arrays of different plasmonic patterns were demonstrated [15], and this helped to somewhat increase the velocity. The currents could be generated by heating with different wavelengths of light in the near IR spectrum shining on a variety of plasmonic structures and rough metal films [14,17,18,19,20]. However, only small particles could be manipulated without causing significant heating. The main reason for that was a prior focus on optical properties of the whole system while ignoring its thermal properties. Later on, it was demonstrated that the thermal conductivity of the substrate plays an important role in the efficiency of the current generation. By adding continuous indium tin oxide (ITO) film to the substrate, the fluid velocity increased, and small particles were able to move with the speeds of up to 2 µm/s [21]. In addition to that, much faster flow can be demonstrated using thermo-plasmonic heating with the generation of a water vapor microbubble, but this requires a pretty high temperature of operation [22,23]. However, in all of these approaches to OFT the manipulated objects were exposed to significant amount of light radiation that could potentially damage cells or interfere with fluorescent imaging. Also, they were done using light coupled through a microscope objective, which made the whole setup inflexible in terms of independent observation and manipulation. Finally, they supported manipulation of small cells or particles on 2D surfaces, while we, for the first time, propose 3D manipulation and movement of much larger objects, truly pushing the limit of micromanipulation while using a very low controlling light power. This was accomplished because we were able to simultaneously optimize optical and thermal properties of our substrate to maximize the performance.

**Figure 1:**
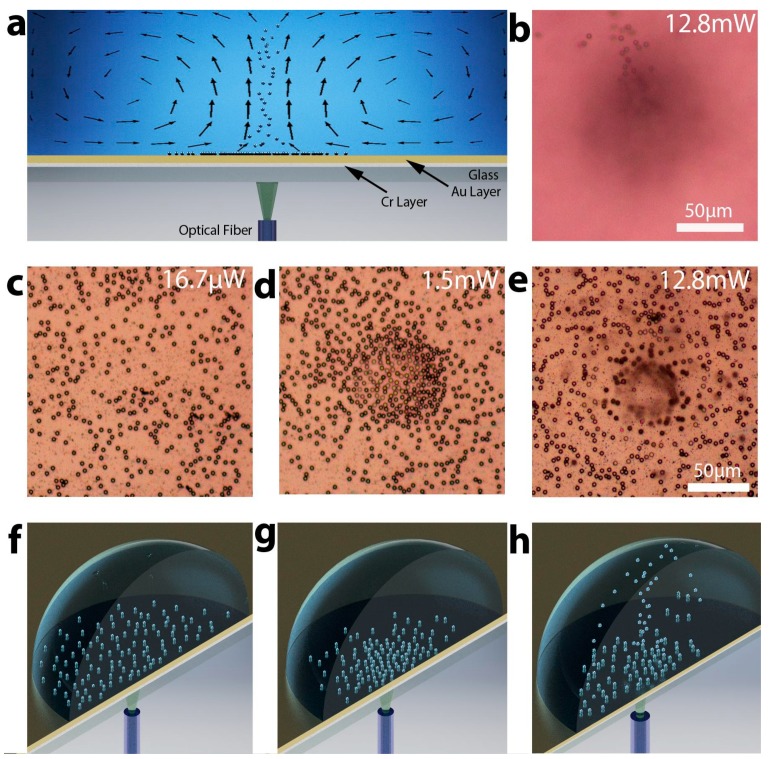
Explanation of opto-fluidic manipulation. (**a**) A 2-D model showing the trapping and levitation of particles using the convective vortices generated by the laser excitation. The arrows show fluid velocity vectors, and the thickness of arrows corresponds to the speed of movement. (**b**) Optical microscope image showing stream of 5 µm polystyrene beads levitated by opto-fluidics. (**c–h**) Optical microscope images and corresponding three-dimensional models of the three regimes of opto-fluidic particle manipulation; (**c**,**f**) low power—no controllable particle movement; (**d**,**g**) medium power—trapping regime; (**e**,**h**) high power—levitation regime. In (**e**) the spot in the center looks out of focus because in that area particles are projected upwards.

Our substrate contains a continuous bi-metal layer (Figure 1a), and this configuration has multiple advantages over traditional thermo-plasmonic structures. In comparison to small plasmonic patterns on transparent substrates, the continuous metal layer allows us to avoid the heating and phototoxicity that occurs due to direct light exposure of cells, while also providing the added advantage of, previously difficult, effective fluorescent imaging. The substrate is composed of a microscope glass slide with sputtered continuous bi-layer of chromium and gold. Light is absorbed by a thin layer of chromium, and the gold layer provides an interface that is biocompatible with cells and blocks the sample from light. Furthermore, the metallic bi-layer is optimized for maximum absorption by supporting a low-quality resonator, providing multiple passes of light through the chromium layer. This metallic bi-layer design can be optimized for various wavelengths by varying the thickness of both metals to optimize compound optical absorption and total thermal conductivity, allowing for the most effective micro-current generation. In this paper we demonstrate the safe and effective biological manipulation at two different wavelengths (532 and 808 nm) and show that the convective current generation can be efficiently supported with the help of the bi-metallic structure. Additionally, we demonstrate that opto-fluidic convection can be applied to several new biological functionalities including cell and particle capturing and levitation, fluid mixing, precise removal of biological material from surfaces, and cell/particle sorting. We also demonstrate that these phenomena can be optimized for ultra-efficient 3D manipulation of micro-scale biological objects, with seven orders of magnitude difference in mass, ranging from objects as tiny as silver nanowires to live organisms as large as an entire *Caenorhabditis elegans* worm.

## 2. Methods

A schematic of the experimental setup is shown in Figure 1a. To manipulate micro-scale particles, we coupled a laser light into an optical fiber that had been cleaved on one end. The fiber was inserted into a fiber holder attached to a micromanipulator allowing XYZ fiber movement. After aligning the cleaved fiber tip underneath the microscope objective, we placed the substrate between the fiber and the objective. We then pipetted a drop of a media containing particles or cells on top of the gold surface. Next, the laser was turned on and light from the optical fiber was shining under the substrate, through the glass layer and chromium. By the time it reached the gold, it was partially attenuated by the chromium layer, it was then reflected by the gold layer and again propagated through the chromium experiencing additional attenuation. The gold layer protects the sample from the excitation light and enhances the attenuation and heat generated by the metallic bi-layer. Since the chromium thickness (5–20 nm) is significantly smaller than thickness of gold (200 nm), the thermal conductivity properties of the metallic bi-layer are dominated by gold. This results in a highly efficient local fluid heating. Continuous laser heating of the substrate locally warms the fluid and it continuously rises to the top of the droplet, this results in the formation of a consistent vortex shaped as a toroid (Figure 1a). We used the horizontal part of the current to push objects towards the center of the heated spot and the vertical part of the current to levitate particles with the stream of warm fluid upwards. This allows trapping relatively large objects, such as live nematodes and levitating a variety of smaller objects, such as red blood cells and micro-beads.

## 3. Results

Here we demonstrate that the intensity of the excitation light can be used to precisely control the fluid velocity and the forces applied to the microscopic objects. Our COMSOL simulations showed that the fluid velocity of the convective vortices is linearly proportional to the temperature of the heat source, while our experiments demonstrated that the temperature of the fluid is linearly proportional to the optical power. Thus, the fluid velocity is also linearly proportional to the optical power. This gave us the ability to easily and effectively adjust the fluid velocity and precisely control the size of the objects that we manipulated.

To study fluid velocity under different heating conditions, we used COMSOL multi-physics software that allows the simultaneous study of heat transfer and fluid dynamics. We simultaneously solve the Navier–Stokes equations and the conservation of energy equation to determine the velocity and the temperature fields. The micro-flow patterns formed by Rayleigh–Benard convection are analyzed.

Equations: Computation fluid dynamics is used to analyze the flow field. We are solving the continuity equation (Equation (1)), Navier–Stokes equations (Equation (2)–(4)), and conservation of energy equation (Equation (5)) for the corresponding initial and boundary conditions. Note that *u*, *v*, and *w* are components of the fluid velocity, *ρ* is density, *T* is the temperature, and *k* is thermal diffusivity. Conditions for Rayleigh–Benard convection are considered.

(1) $$\frac{\partial u}{\partial x}+\frac{\partial v}{\partial y}+\frac{\partial w}{\partial z}=0$$

(2) $$\frac{\partial u}{\partial t}+u\frac{\partial u}{\partial x}+v\frac{\partial u}{\partial y}+w\frac{\partial u}{\partial z}=-\frac{1}{\rho }\frac{\partial \delta }{\partial x}+f_{x}+v\left(\frac{\partial^{2}u}{\partial x^{2}}+\frac{\partial^{2}u}{\partial y^{2}}+\frac{\partial^{2}u}{\partial z^{2}}\right)$$

(3) $$\frac{\partial v}{\partial t}+u\frac{\partial v}{\partial x}+v\frac{\partial v}{\partial y}+w\frac{\partial v}{\partial z}=-\frac{1}{\rho }\frac{\partial \delta }{\partial y}+f_{y}+v\left(\frac{\partial^{2}v}{\partial x^{2}}+\frac{\partial^{2}v}{\partial y^{2}}+\frac{\partial^{2}v}{\partial z^{2}}\right)$$

(4) $$\frac{\partial w}{\partial t}+u\frac{\partial w}{\partial x}+v\frac{\partial w}{\partial y}+w\frac{\partial w}{\partial z}=-\frac{1}{\rho }\frac{\partial \delta }{\partial z}+f_{z}+v\left(\frac{\partial^{2}w}{\partial x^{2}}+\frac{\partial^{2}w}{\partial y^{2}}+\frac{\partial^{2}w}{\partial z^{2}}\right)$$

(5) $$\rho C_{P}\left(\frac{\partial T}{\partial t}+u\frac{\partial T}{\partial x}+v\frac{\partial T}{\partial y}+w\frac{\partial T}{\partial z}\right)=-k\left(\frac{\partial^{2}T}{\partial x^{2}}+\frac{\partial^{2}T}{\partial y^{2}}+\frac{\partial^{2}T}{\partial z^{2}}\right)$$

The onset of natural convection is determined by the Rayleigh number Ra. For thermal convection due to heating from below, Ra $=\frac{\rho_{0}\beta g\Delta TL^{3}}{\alpha \mu }$, where $\rho_{0}$ is the reference density, typically picked to be the average density of the medium, *g* is the local gravitational acceleration, *β* is the coefficient of thermal expansion, Δ*T* is the temperature difference across the medium, *L* is the characteristic length-scale of convection, *α* is a thermal diffusivity, and *µ* is the dynamic viscosity. Preliminary data based on COMSOL simulations show the velocity of the observed convection exhibits an approximately linear relationship with the laser power. Arrows representing velocity vectors simulated using COMSOL are shown in Figure 1a. We extracted the fluid pattern from the simulations and combined it with the illustration of the setup to simplify the understanding of the current structure. The simulations of the laser light-controlled currents were conducted for substrate temperatures ranging from 30 to 50 °C. In simulations at all temperatures, the current structure was very similar to the one shown in Figure 1a. The main difference was that all velocities were proportionally scaling up. While fluid velocity measured in the center of the droplet was ~1.2 µm/s at 30 °C, it reached 6.5 µm/s at 50 °C.

### 3.1. The Substrate Optimization, Fabrication, and Characterization

**Figure 2:**
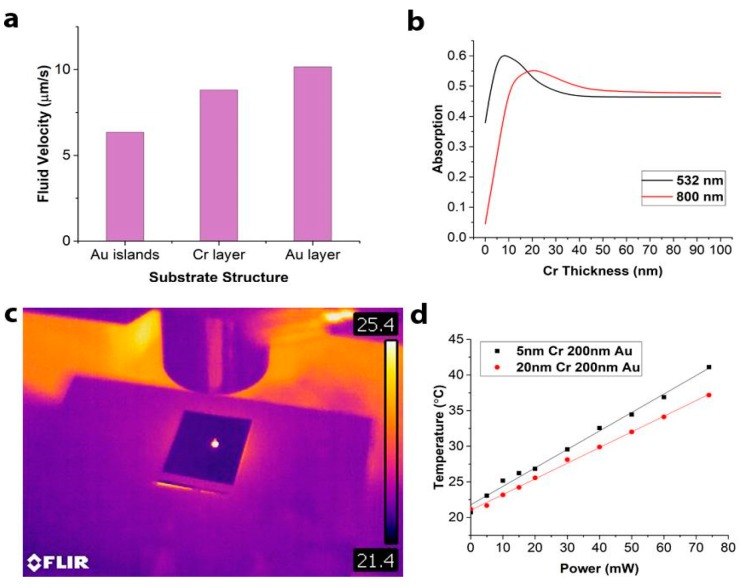
The substrate optimization. (**a**) COMSOL simulations were conducted to evaluate the most efficient substrate structure. (**b**) Finite difference time domain (FDTD) simulation results for substrate absorption optimization for 532 and 800 nm wavelengths varying chromium thickness from 0 to 100 nm while holding gold thickness constant at 200 nm. (**c**) FLIR IR camera imaging was used to measure the maximum temperature in a 1 µL drop of fluid. (**d**) Experimentally measured temperature increase while testing two substrates with 532 nm light with power ranging from 0 to 80 mW.

In contrast with previous studies, which used different types of patterned plasmonic structures and nanoparticles, we chose continuous metallic bi-layer. We found out from our COMSOL simulations that the thermal conductivity of the substrate greatly affects the efficiency of the current generation and its velocity. Figure 2a demonstrates that by heating three differently structured substrates (continuous gold layer, continuous chromium layer, and gold islands) to the same temperature, we observe a noticeable difference in the fluid velocity. Since gold has the highest thermal conductivity, the resulting convective current induced in the fluid is the most powerful. A continuous layer of chromium is less efficient due to its lower thermal conductivity and gold islands that do not form a continuous thermally-conductive layer were observed to be the least efficient. Therefore, the optimal structure should be continuous and have high thermal conductivity, preferably closer to gold.

After determining the requirements for the thermal conductivity of the substrate, the next step was to take into consideration its optical and biocompatibility properties. A metallic bi-layer was chosen as a substrate because no single metal could simultaneously satisfy all our requirements: biocompatibility, high thermal conductivity, and high optical absorption for visible/IR wavelengths. Chromium was chosen for one of the layers for its high absorption for chosen wavelengths (532 and 800 nm) and gold was selected for its biocompatibility, thermal conductivity, and reflective properties. The individual metal thicknesses of the bi-layer were then studied to optimize overall performance. To optimize the optical absorption of the bi-layer we conducted finite difference time domain OptiFDTD simulations. The goal was to create a substrate that has opaque metallic bi-layer efficiently absorbing controlling light and isolating the sample from any light exposure. The gold layer thickness was chosen to be 200 nm for optimal reflection, while the thickness of the chromium layer was varied between 5 and 200 nm in the simulations (Figure 2b). Chromium and gold were both modeled as Lorentz–Drude dispersive materials. Optimization was conducted for wavelengths 532 and 800 nm because of wide availability of low-cost lasers with those wavelengths. In simulations the beams were linearly polarized, had a Gaussian profile, were emitting continuously, and were perpendicular to the substrate. 

The absorption by bi-metallic substrate at both wavelengths with respect to the chromium thickness is plotted in Figure 2b. Since gold is highly reflective at both wavelengths, adding a layer of 200 nm of gold on top of chromium effectively doubles the propagation length of the light in the chromium layer. The absorption of the bi-layer is the highest for 5 nm of chromium at 532 nm and 20 nm of chromium at 800 nm. Interestingly, the optimized absorption by the layered structure was found to be 25%–30% higher than absorption by a single thick layer of Cr. This is the result of multiple reflections in the low-quality factor plasmonic resonator consisting of a metal bi-layer on a glass substrate. 

Based on the results of the simulations, we fabricated substrates optimized for 532 and 808 nm lasers (5nm-Cr/200nm-Au and 20nm-Cr/200nm-Au, respectively), with the bi-layers deposited on glass slides by sputtering. After fabrication, the performance of the substrates was experimentally evaluated. All temperature measurements were conducted using a FLIR thermal camera. It was demonstrated that under local light exposure from an optical fiber, both substrates were able to efficiently absorb light and heat a 1 µL drop of water placed on top of the substrate (Figure 2c), reaching a maximum equilibrium temperature within 1–2 minutes after beginning of the experiment. As the power of the laser beam was increased from 0 to 80 mW, the temperature increased linearly from 20 to 40 °C (Figure 2d). This result, when combined with the linear relationship between the temperature and the fluid velocity vectors observed in COMSOL simulations, allowed us to infer a linear relationship between the fluid velocity in the generated convective vortex and the laser power.

Additionally, this result further demonstrated that an increase of 4 mW of the laser power results in a corresponding water temperature increase of 1 °C. Finally, it was experimentally confirmed that, similarly to the simulations shown in Figure 2b, the absorption at 532 nm is more efficient for chromium thickness of 5 nm comparing to 20 nm (Figure 2d), though both of them perform quite well.

### 3.2. Sample Manipulation Using Optimized Substrates

**Figure 3:**
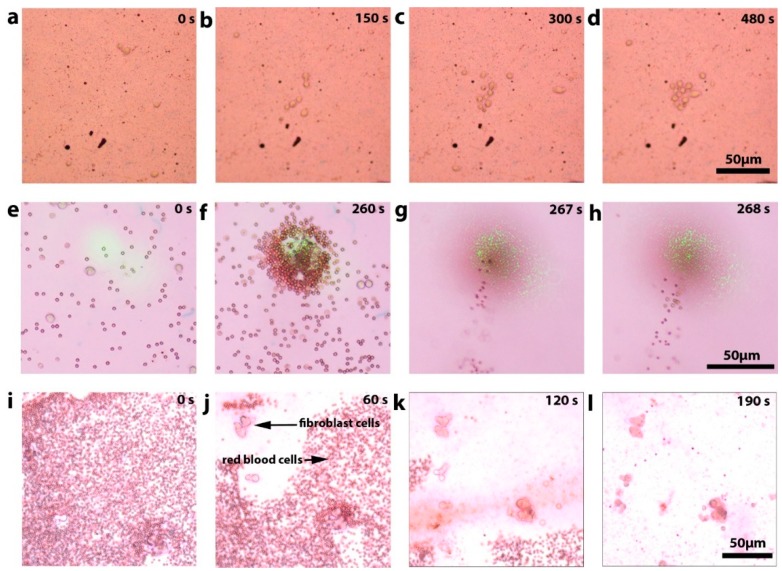
Experimental demonstration of several opto-fluidic manipulation regimes. (**a**–**d**) Trapping of fibroblast cells during 480 second period. (**e**–**h**) Trapping of heavy fibroblast cells simultaneously with the projection of lighter red blood cells. (**e**,**f**) Microscope imaging plane of the substrate, (**g**,**h**) imaging above the substrate demonstrates stream of red blood cells being levitated. (**i**–**l**) Targeted “pressure washing” removing light red blood cells from the surface leaving in place heavy fibroblast cells. Optical fiber scans under the whole portion of the substrate visible in the field of view selectively removing one type of the cells from the surface.

After optimizing the substrate, we demonstrated several new applications of 3D opto-fluidic manipulation to biological specimens including cell accumulation/trapping, projection, separation/filtering, followed by a viability study proving the safety of this approach to living cells. In the following experiments, we used human fibroblast cells suspended in a cell growth medium. The cells were treated with a 0.25% (w/v) Trypsin—0.53 mM ethylenediaminetetraacetic acid (EDTA) solution helping to temporarily keep the cells from bonding to the gold surface during experiments. We demonstrated the operation of the trapping regime at a controlling power of 15 mW using the 20 nm-Cr/200 nm-Au substrate optimized for 808 nm wavelength (Figure 3a–d). For this experiment a 5 µL drop of cell medium containing live fibroblast cells was placed on the substrate, and the optical fiber was aligned under an area of the substrate that initially contained no cells (Figure 3a). After 480 seconds, ten fibroblast cells were captured, and some of them from a distance greater than 250 µm (the field of view of the microscope).

To demonstrate the projection regime, we used a laser power of 47 mW applied to 5 nm-Cr/200 nm-Au substrate optimized for 532 nm wavelength. The sample was pipetted from a mixture of 1 µL of human blood and 1 mL of the medium containing fibroblast cells. The induced convection vortices were strong enough to levitate red blood cells vertically from the surface. After 260 seconds, we observed that both red blood cells and fibroblast cells were trapped in the center of the excitation area and there was a continuous stream of red blood cells projected upward (Figure 3e–h).

In the next set of experiments, it was observed that if we scan the optical fiber under the substrate (within the field of view), while operating in a high-power projection regime, we were able to successfully levitate and remove red blood cells from the surface without moving the fibroblast cells or trapping red blood cells (Figure 3i–l). The laser power used in these experiments was the same as in the previous experiment (47 mW). When optical fiber moves, the toroid current pattern does not have enough time to form, and the vertical water movement simply projects the red blood cells off the surface, like a miniature “pressure washing” system. These tree regimes—trapping, levitation, and pressure washing can be in parallel and differently applied to different types of particles with applications in trapping, mass-based filtrations/separations, measurements of masses of individual cells, and sensing applications.

**Figure 4:**
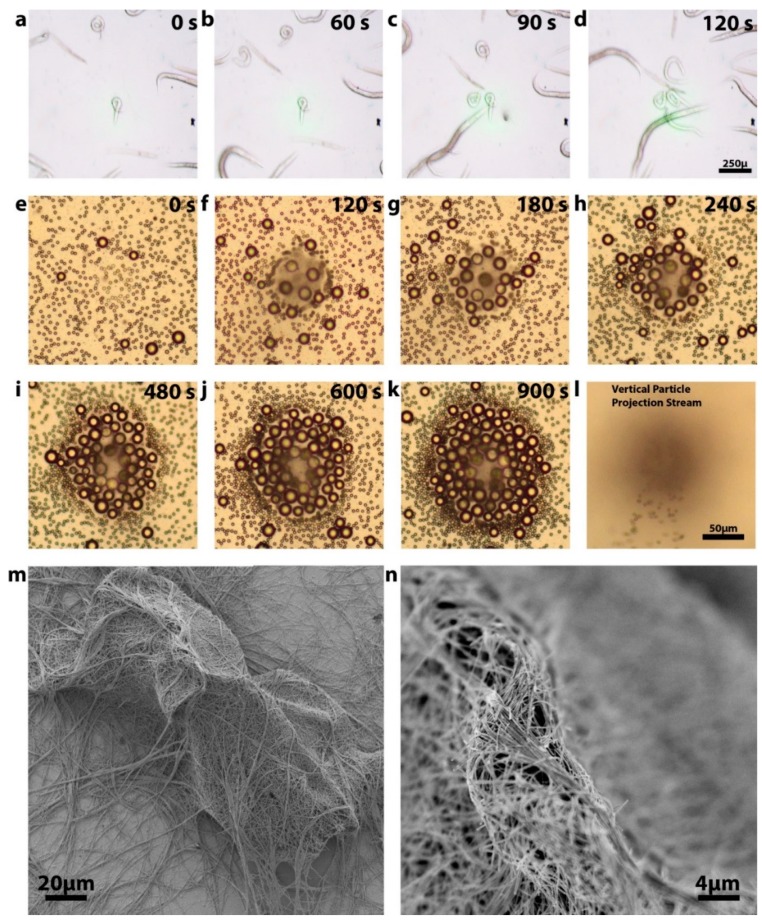
Demonstration of the extraordinary dynamic range of opt-fluidic manipulation and application to the assembly of complex structures. (**a**–**d**) Capturing live worms using 40 mW power. Worm length varied from 50 to 200 um. (**e**–**l**) Selective capturing of large particles and their multi-layer assembly. (**m**,**n**) A multi-layer silver nanowire structure built using trapping and levitation.

Finally, the following experiments were conducted to demonstrate the extraordinary dynamic range of masses that could be manipulated using this technology, along with showing that this approach can also be used to build complex, multi-layer structures (Figure 4). First, we trapped live *C. elegans* worms using a 35 mW laser power (Figure 4a–d). The worms were still active after the manipulation, showing that the temperature increase did not damage them. The live worms are the largest reported living organisms manipulated using light. They have an average length of 1 mm and width of 50 µm. In contrast to the macroscopic worms, we show that the same technique can be used to manipulate objects as small as silver nanowires which have masses seven orders of magnitude smaller than that of worms (Figure 4m,n). Using our opto-fluidic manipulation we were not only able to capture silver nanowires from the solution but demonstrated the ability to levitate them and build multi-layer structures. In future this can be used for building more complex 3D nano-scale structures without the use mechanical manipulators. Additionally, we also demonstrated that our approach can be used for selective size-based trapping and assembly (Figure 4e–l). We were able to separate 20 µm polystyrene micro-beads from their mixture with 5 µm beads by trapping larger particles and projecting away smaller ones. The 20 µm beads assemble in multi-layer structure that can be potentially used for building photonic crystals and other complex ordered assemblies.

### 3.3. Cell Viability

**Figure 5:**
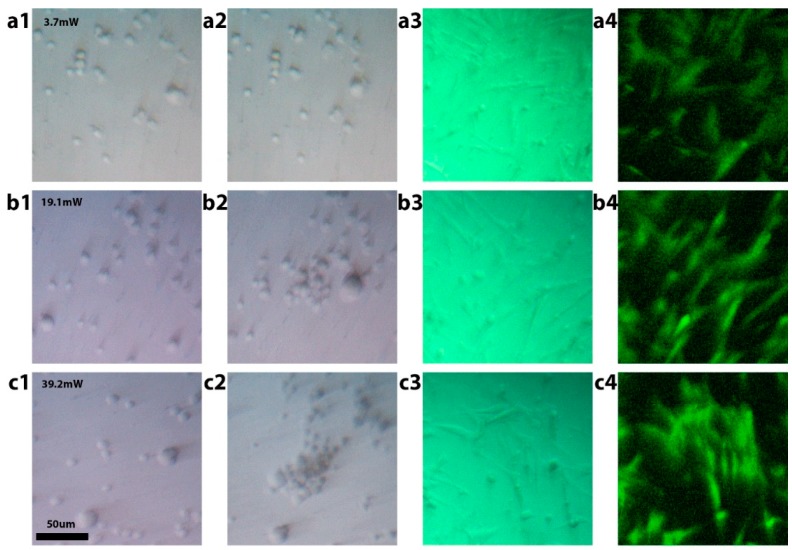
Demonstration of fibroblast cell viability after opto-fluidic manipulation. (**a1,a2**) Before and after low-power manipulation at 3.7 mW. (**b1,b2**) Before and after medium-power trapping at 38 mW. (**c1,c2**) Before and after high-power projection regime at 78 mW. (**a3,b3,c3**) Bright-field and (**a4,b4,c4**) fluorescent images of cells after 24 hours of incubation.

To test the biocompatibility of the technology, a series of cell viability tests were conducted for different power regimes used in cell manipulation experiments (Figure 5). To ensure all cells used were studied under the same conditions, we integrated the substrate with a PDMS chip containing multiple identical wells. Each well was used to test cell viability after manipulation using one of the regimes, in addition to wells used for control experiments without any manipulations. The fibroblast cells were manipulated in low-power regime (3.7 mW), medium-power trapping regime (38 mW), and the high-power projection regime (78 mW) (Figure 5a1,a2,b1,b2,c1,c2). The cells in each well were manipulated for 150 seconds, after which, the whole chip was returned into an incubator, where the cells were cultured for 24 hours. During that time, the cells bonded to the gold surface and started growing. Bright-field microscopy demonstrated that the cells looked healthy and had an expected morphology (Figure 5a3,b3,c3). We then used a live/dead cell imaging kit to stain live cells with green-fluorescence-emitting dye, and dead ones with red-emitting dye. It was confirmed using fluorescence imaging that the cells appeared healthy and emitted a green fluorescence signal (Figure 5a4,b4,c4). After a detailed examination, on average, less than one dead cell was observed per well, matching the results from the control well that was not exposed to any manipulations. This result confirmed that all regimes of opto-fluidic manipulation were biocompatible and did not decrease cell viability.

## 4. Conclusions

To summarize, we have demonstrated for the first time several new regimes of opto-fluidic manipulation. We showed the trapping of objects with the extraordinary seven orders of magnitude dynamic range of masses, from microscopic nano-wires to macroscopic live *C. elegans* worms. Furthermore, our studies have shown that we not only captured, but levitated, filtrated, and “pressure washed” a variety of objects. On top of this, we have observed, through cell viability testing, that this technology can be safely used, not only for capturing but also the levitation and separation of cells and other biological organisms. We also show that this method can be customized for different wavelengths of light. This opens the door for consistent, reproducible, and easily fabricated devices that can be implemented across a wide range of fields that require gentle and powerful biomanipulation of objects and cells, and small live organisms. We suggest that some of the potential applications might include filtering different types of cells, separation of bacteria or circulating tumor cells from biological samples. Additionally, capturing worms using our approach is very attractive, since all traditional mechanical ways to move the worms around require use of sharp metal tips and other objects that damage worms, while optical tweezers would require extremely high power that would kill them. At the same time, our approach allows gentle movement of live worms to a needed location for further imaging and analysis.
